# United States

**Published:** 1896-08

**Authors:** 


THE UNITED STATES VETERINARY COLLEGE announcement for the session of '96 and '97 announces its return to the three-year schools of six months each. Dr. C. H. Ford, Professor of Dental Surgery, and one of the hospital surgeons as well as one of the trustees of the institution, has severed his connection therewith, and as hospital surgeon is succeeded by Andrew C. Seacord, graduate of the Class of '96, who will also fill the Chair of Dental Surgery and Demonstrator of Anatomy. Dr. J. H. Adamson has also severed his connection with the school as Professor of Anatomy, and this subject will be taught by the Dean, C. Barnwell Robinson, during the coming year. John B. Daish, who last year filled the Professorship of Botany and Zoölogy, is not associated with the school during the coming year. Dr. Alexander Dunn, who held the Chair of Materia Medica and Toxicology, has been succeeded by William A. Hedrick as instructor in materia medica, associated with his former course of chemistry.

Fifteen students were registered during the term of '95 and '96. The matriculation examination is the same as recommended by the Association of Faculties of North America.

